# Cumulative inflammatory burden of metal mixtures is associated with central obesity, cardiovascular disease, and mortality: findings from NHANES

**DOI:** 10.3389/fcell.2026.1717247

**Published:** 2026-02-18

**Authors:** Yuanhao Feng, Qing Chen, Jiawei Peng, Zhihao Ma, Yiduo Bai, Wenjun Liu, Jijun Wu

**Affiliations:** 1 Department of General Surgery, The Sihui People’s Hospital, Zhaoqing, China; 2 Orthopedic of Department, Shenzhen Third People’s Hospital, Shenzhen, Guangdong, China; 3 Department of Vascular Surgery, Guangdong Provincial Key Laboratory of Major Obstetric Diseases, Guangdong Provincial Clinical Research Center for Obstetrics and Gynecology, The Third Affiliated Hospital, Guangzhou Medical University, Guangzhou, Guangdong, China; 4 Department of Interventional Radiology, Binhaiwan Central Hospital of Dongguan, Dongguan, Guangdong, China; 5 Department of Interventional Radiology, Zhongshan Torch Development Zone People’s Hospital, Zhongshan, Guangdong, China

**Keywords:** metal mixtures, inflammatory burden, cardiovascular disease, central obesity, NHANES, mortality risk

## Abstract

**Background:**

The exposure of heavy metals is a serious environmental risk factor for cardiometabolic health. However, the association of cumulative inflammatory load of metal mixtures with cardiovascular disease (CVD) and mortality remain incompletely characterized. This study aimed to evaluate the associations of a metal mixture inflammatory index (MMII) with CVD and mortality, and to examine whether central obesity measured by weight-adjusted waist index WWI statistically mediates the association of MMII with CVD in an exploratory cross-sectional study.

**Methods:**

Data were obtained from 7 cycles of the National Health and Nutrition Examination Survey (NHANES 2005–2018) with mortality follow-up through 2019. A total of 11,577 adults aged ≥20 years with complete urinary metal, inflammatory markers, CVD, and WWI data were included. Using inductively coupled plasma mass spectrometry (ICP-MS), nine metals’ urinary concentrations were measured. MMII was calculated using reduced rank and stepwise regression based on inflammation biomarkers. Linear association of MMII with CVD was examined using logistic regression and Cox proportional hazards models, along with RCS and exploratory mediation analyses for associations with prevalence and mortality.

**Results:**

Elevated MMII levels were noticeably associated with increased odds of CVD (OR = 2.79, 95% CI: 1.81–4.29). Estimates from mediation analyses further indicated that WWI statistically explained 11.4% of the effect observed. Elevated MMII was associated with a higher risk of all-cause mortality (HR = 2.39, 95% CI: 1.69–3.38), without significant association with cardiovascular mortality.

**Conclusion:**

Elevated levels of MMII reflect the burden of metal mixtures and are linked to central obesity, cardiovascular disease (CVD), and all-cause mortality.

## Introduction

The world is facing a serious public health hazard to humans and the ecosystem, due to heavy metals exposure, prompted by rapid industrialization and urbanization. Industrial effluents, mining, agriculture, and fuel combustion are continuously releasing heavy metals to air, water, and soil beyond their natural self-purification (Zeng et al., 2023). The major exposure routes of humans to these toxicants are inhalation of polluted air, ingestion of contaminated food and water, and skin absorption (Jomova et al., 2025; Zhao et al., 2023). Heavy metals are certainly persistent and resist biological degradation. As a result, they can bioaccumulate on human tissues. Furthermore, they are related to long-term toxic effects (Ferreira et al., 2022). Epidemiologic evidence has shown that cadmium or lead exposure increases cardiovascular risk and/or mortality (Liu J. et al., 2025; Lamas et al., 2023). Mining activity is always an environmental hazard thus, more recently, expanded mining activities increased human exposure to emerging metals like tungsten and uranium. However, the mortality consequences of which remain poorly known (Wang et al., 2025).

Importantly, authentic experiences of exposure seldom revolve around a single metal. That is to say, people usually have multiple metals, or even trace elements, to which they may be exposed at once, and These may exert additive, synergistic, or even antagonistic effects (Yan et al., 2022). Consequently, a rising number of epidemiologic studies have been adopting mixture-oriented approaches for evaluating cardiovascular outcomes under co-exposure scenarios (Occelli et al., 2020). For example, recent studies report that multi-element exposure profiles are linked to the prevalence and severity of acute myocardial infarction, suggesting that co-exposure is relevant for cardiovascular risk (Sun et al., 2025). Simultaneously, larger body of evidence syntheses show that metal-mixture analyses (e.g., BKMR, WQS, and related methods) often identify joint associations of combined metal exposures with risk factors and CV outcomes, including NHANES-based studies (Yim et al., 2022; Zhang et al., 2021). Despite rising interest in mixture models, much less is known about the degree to which multi-metal exposures contribute to cardiovascular risk through systemic inflammation at the population level.

low-grade systemic inflammation is a major biological mechanism through which environmental toxicants lead to chronic diseases and shortened life spans. The chronic stimulation of immune cells and excessive accumulation of pro-inflammatory cytokines contribute to vascular injury, insulin resistance, and oxidative stress (Medrano-Bosch et al., 2023; Zha et al., 2022). Experimental and epidemiological studies indicate that combined exposure to lead and cadmium induces oxidative stress and impairs vascular function (Lamas et al., 2023). Several hematological indices, including neutrophil-to-lymphocyte ratio (NLR), platelet-to-lymphocyte ratio (PLR), and systemic immune-inflammation index (SII), have been applied to quantify inflammatory burden (Yontar and Mutlu, 2024; Ma et al., 2024). However, in population-based studies, evidence on the role of cumulative metal exposure in systemic inflammation is scarce. In a recent study, the Metal Mixture Inflammatory Index (MMII) was proposed as a composite biomarker for the data-driven quantification of the systemic inflammatory potential of multiple urinary metals (Wang et al., 2024). Nevertheless, the MMII’s role in the etiology of CVD is still unknown.

Another significant risk factor for cardiometabolic diseases is chronic low-grade inflammation of central obesity. C-reactive protein (CRP) is associated with the inflammatory response condition and obesity (Li et al., 2020). Weight-relate Waist Index (WWI), a new indicator of anthropometric measurements, is a superior measure of abdominal obesity independent of body weight, unlike other indices like body mass index (BMI) (Kim et al., 2022). Insulin resistance and cardiovascular disease (CVD) have been tightly linked to elevated WWI (Liu S. et al., 2025). Given the close relationship between systemic inflammation and central obesity, WWI was assessed for its potential as a statistical mediator in an exploratory context. Despite these insights, few studies have evaluated the associations between heavy metal mixtures and systemic inflammation, central obesity, and CVD concurrently. Whether MMII is related to CVD risk in the general population remains uncertain, as does whether WWI is a statistical mediator. Addressing these gaps is essential for understanding the interplay between environmental exposures and cardiometabolic health. The primary contributions of our research/article are threefold. As far as we aware, this is the first study to investigate the association of the MMII and CVD in a nationally representative U.S. population. Secondly, we assess WWI as a new statistic mediatory in the metal-related inflammatory burden and cardiovascular outcomes association. Third, we assess metal mixture rather than metal by looking at cumulative inflammatory impact which reflects the environmental exposure more realistic. Collectively, these elements emphasize the public health importance of metal mixtures and the need to adapt environmental, inflammatory, and anthropometric perspectives in environmental epidemiology.

## Materials and methods

### Study population

The National Health and Nutrition Examination Survey (NHANES) is a continuous, nationally representative survey of the civilian, non-institutionalized U.S. population conducted by the Centers for Disease Control and Prevention (CDC) using a complex, multistage probability sampling design (Zhao et al., 2024; Peng et al., 2023). The survey design and data collection methods have been outlined in detail (Terry et al., 2024).

**FIGURE 1:**
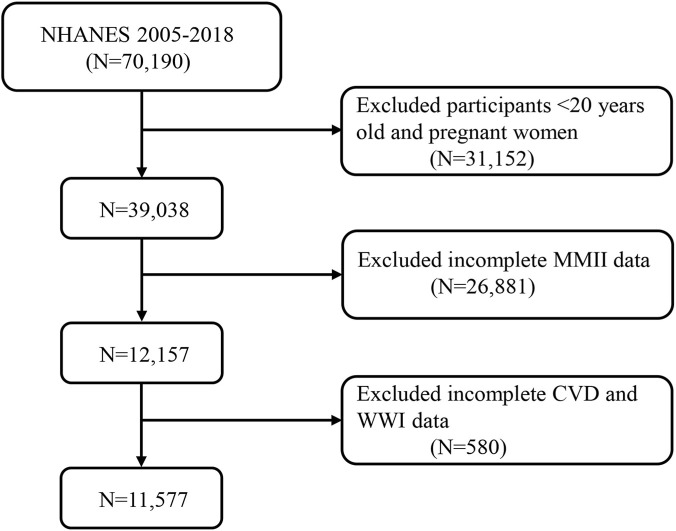
A flow diagram of eligible participant selection in the National Health and Nutrition Examination Survey. Abbreviation: MMII, Metal Mixture Inflammatory Index; CVD, Cardiovascular Disease; WWI, weight-adjusted waist index.

For this study, data from seven NHANES cycles (2005–2018) were pooled, which resulted in a sample of 70,190. The study excluded applicants aged below 20 years and who were pregnant (n = 31,152). Of the remaining participants, 26,881 (38.3%) were excluded due to missing data required for MMII construction. Most of these exclusions occurred because urinary metal measures and inflammatory biomarkers only exist in NHANES subsamples a. Another 580 were excluded for missing cardiovascular disease (CVD) or weight-adjusted waist index (WWI). The final analytic sample included 11,577 adults (Figure 1). Informed consent was obtained from all participants in the study, which conforms to all National Center for Health Statistics Research Ethics Review Board protocols.

### Construction of the metal mixture inflammatory index (MMII)

The Metal Mixture Inflammatory Index (MMII) was constructed to evaluate the cumulative inflammatory contribution of combinations of urinary metals through a data-driven approach. Urinary concentrations of nine metals (Mercury, Cadmium, Cobalt, Molybdenum, Lead, Antimony, Thallium, Tungsten, Uranium) were included as exposure variables. Adjustments for urine dilution were made through urinary creatinine (Supplementary Table S1). Prior to statistical analyses, all metal concentrations were log-transformed to reduce right skewness, then standardized to z-scores (mean = 0, SD = 1).

The standardized urinary metal concentrations were used as predictors and the four systemic inflammatory index biomarker concentrations (CRP, NLR, PLR, and SII) were response variables (Lucas et al., 2014). Reduced rank regression (RRR) was applied for this purpose. This RRR component was retained owing to its ability to explain the maximum variability in the inflammatory biomarker set. It represents the metal mixture pattern most strongly associated with low-grade systemic inflammation.

Subsequently, stepwise linear regression was conducted to achieve better interpretability and parsimony, with the retained RRR component score as the dependent variable and standardized metal concentrations as independent variables (significance level P < 0.10 for variable retention). The retained metals in the final model were weighted by their regression coefficients. Individual MMII scores were calculated as a weighted sum of the standardized concentrations of the selected metals using the following formula: *MMII = Σ (βᵢ × Zᵢ). Zᵢ* indicates the standardized concentration of metal i and βᵢ is the regression coefficient of metal i. Higher values of MMII suggest higher estimated inflammatory burden due to multi-metal exposure (Supplementary Table S3).

RRR models reflect the cumulative systemic inflammatory capacity that results from exposure to many heavy metals. The low-grade systematic inflammation RRR metal exposure pattern was retained as the first factor resulting from RRR (Additional file 3) which suggested strong correlation. The heavy metals discovered through these analyses were assigned regression coefficients weights, which on summation made up the MMII score. The Material Mediated Inflammation Index estimates the inflammatory potential of a person’s mixed metal exposure, with higher scores being more proinflammatory. After this, the weighted multivariate COX regressions were performed to evaluate whether standardized MMII was associated with mortality, which allowed us to conduct a complete analysis of metal mixture-mortality association.

### Assessment of WWI

The WWI is a indicator of abdominal obesity that is independent of weight. The waist-to-weight ratio is calculated by dividing a person’s waist circumference (WC in cm) by the square root of body weight (in kg) (Park et al., 2018). The NHANES website offers “Body Measures” data, which include WC and weight data. The manuals for NHANES provide information on methods WC was measured with measuring tape at specific anatomic sites. Greater central adiposity is indicated by higher WWI values.

### Covariates

The current study’s covariates were predominantly classified into the two categories, namely, demographic status and medical conditions. The demographic covariates refer to age (years), gender (male/female), race (Mexican American/Other Hispanic/Non-Hispanic White/Non-Hispanic Black/Other) and education status (Below high school/High School or above). Presence of diabetes and hyperlipidemia (yes/no) (Supplementary Table S2).

### Outcome ascertainment and follow-up of mortality

We used NHANES publicly available linked mortality files as of 31 December 2019 (https://www.cdc.gov/nchs/data-linkage/mortality-public.htm), to determine the vital status and cause of death in the follow-up population. The information used in this study is retrieved from de-identified public databases. The definition of cardiovascular disease (CVD) prevalence was based on self-reported physician diagnosis of coronary heart disease, myocardial infarction, angina, heart failure or stroke during the NHANES interview. The evaluation of mortality outcomes was performed through probabilistic linkage with the public-use linked mortality files based on the NHANES. The last follow-up date was set at 31 December 2019. Follow-up time was calculated from the date of examination in the NHANES through the death date or follow-up end, whichever applied first. Participants without mortality linkage information were excluded from mortality analyses.

### Statistical analysis

The weighted means were used for continuous variables and weighted frequencies and percentages were used for categorical variables. Cox proportional hazards models were used to evaluate the associations of MMII and CVD status with all-cause mortality, with hazard ratios (HRs) and 95% confidence intervals (CIs) estimated. Follow-up time was calculated from the NHANES examination date to the date of death or the end of follow-up (31 December 2019), whichever occurred first. Logistic regression models were used to examine associations between MMII and prevalent CVD. To identify possible nonlinearities, restricted cubic spline analyses were performed. The median value of each Tertile was modeled as a continuous variable to conduct trend tests by MMII quartiles. To decrease skewness in distribution, logarithmic transformation of heavy metal concentrations and systemic inflammation marker level was done before association.

The associations between MMII and WWI were assessed using linear regression models. Exploratory mediation analyses estimated what proportion of the MMII and CVD association is statistically attributable to WWI, decomposing the whole association into direct and indirect parts. The mediation results of the CVD analyses should be considered exploratory and hypothesis-generating rather than causal given their cross-sectional nature. At the moment, we cannot use survey weights in mediation analysis. This should be considered when interpreting our findings.

Due to the cross-sectional nature of the CVD analyses, results from these mediation analyses which were conducted under standard assumptions of causal ordering should be considered exploratory and hypothesis-generating rather than causal. All descriptive analyses and primary regression models were conducted using NHANES sampling weights, strata, and primary sampling units to account for the complex survey design. Urinary metal analyses incorporated the appropriate subsample weights as recommended by NHANES analytic guidelines. Mediation analyses were performed without survey weights due to current methodological limitations, and results should be interpreted cautiously.

## Results

### Baseline characteristics

A total of 11,577 participants were included in the present analysis, which corresponds to a weighted population of 68,671,127 U.S. adults, among whom 5,209,576 (7.6%) had cardiovascular disease (CVD). Participants who had CVD were older than those without CVD. Similarly, CVD participants were more likely to be male and more likely to identify as Non-Hispanic White (All P < 0.001). Individuals with CVD had lower levels of education and less favourable socioeconomic profiles.

The proportion of diabetes (37% versus 12%) and hyperlipidemia (88% versus 66%) was found to be significantly greater among CVD patients. Furthermore, the participants with CVD had significantly higher levels of MMII and WWI, as well as less-favorable metabolic and anthropometric characteristics (all P < 0.001) (Table 1).

**TABLE 1:** Baseline characteristics of all participants were stratified by CVD, weighted.

Characteristic	Overall, N = 68,671,127 (100%)	Non-CVD, N = 63,461,551 (92%)	CVD, N = 5,209,576 (7.6%)	P Value
No. of participants in the sample	11,577	10,423	1,154	-
Age (%)	​	​	​	**<0.001**
*20–40*	26,327,957 (38%)	26,016,011 (41%)	311,946 (6.0%)	​
*41–60*	26,180,585 (38%)	24,678,223 (39%)	1,502,362 (29%)	​
*>60*	16,162,585 (24%)	12,767,316 (20%)	3,395,269 (65%)	​
Sex (%)	​	​	​	**0.002**
*Female*	34,582,779 (50%)	32,271,079 (51%)	2,311,700 (44%)	​
*Male*	34,088,348 (50%)	31,190,472 (49%)	2,897,876 (56%)	​
Race (%)	​	​	​	**<0.001**
*Non-Hispanic White*	46,084,704 (67%)	42,197,280 (66%)	3,887,424 (75%)	​
*Non-Hispanic Black*	7,589,272 (11%)	6,954,405 (11%)	634,867 (12%)	​
*Other*	9,094,676 (13%)	8,650,969 (14%)	443,707 (8.5%)	​
*Mexican American*	5,902,476 (8.6%)	5,658,897 (8.9%)	243,579 (4.7%)	​
Married/Live with partner (%)	​	​	​	**0.009**
*no*	24,747,258 (36%)	22,631,762 (36%)	2,115,496 (41%)	​
*yes*	43,915,007 (64%)	40,825,384 (64%)	3,089,622 (59%)	​
Education level (%)	​	​	​	**<0.001**
*Below high school*	11,132,528 (16%)	9,836,596 (16%)	1,295,932 (25%)	​
*High School or above*	57,519,583 (84%)	53,607,385 (84%)	3,912,197 (75%)	​
PIR (%)	​	​	​	**<0.001**
*Poor*	13,316,911 (21%)	12,018,825 (20%)	1,298,086 (27%)	​
*Not Poor*	50,439,618 (79%)	46,885,827 (80%)	3,553,790 (73%)	​
Diabetes (%)	​	​	​	**<0.001**
*no*	59,251,884 (86%)	55,949,697 (88%)	3,302,187 (63%)	​
*yes*	9,419,243 (14%)	7,511,854 (12%)	1,907,389 (37%)	​
Hyperlipidemia (%)	​	​	​	**<0.001**
*no*	22,324,588 (33%)	21,714,541 (34%)	610,048 (12%)	​
*yes*	46,344,683 (67%)	41,745,154 (66%)	4,599,529 (88%)	​
MMII (mean (SE))	0.09 (0.25)	0.08 (0.25)	0.20 (0.26)	**<0.001**
MMII, tertile (%)	​	​	​	**<0.001**
*T1*	22,891,047 (33%)	21,915,885 (35%)	975,162 (19%)	​
*T2*	22,870,414 (33%)	21,254,876 (33%)	1,615,537 (31%)	​
*T3*	22,909,667 (33%)	20,290,790 (32%)	2,618,877 (50%)	​
WWI (mean (SE))	10.95 (0.82)	10.91 (0.81)	11.48 (0.76)	**<0.001**
WWI, tertile (%)	​	​	​	**<0.001**
*T1*	22,887,567 (33%)	22,261,574 (35%)	625,993 (12%)	​
*T2*	22,892,917 (33%)	21,408,440 (34%)	1,484,477 (28%)	​
*T3*	22,890,643 (33%)	19,791,536 (31%)	3,099,107 (59%)	​

Mean (SE) for continuous variables: the P value was calculated by the weighted Students T-test. Percentages (weighted N, %) for categorical variables: the P value was calculated by the weighted chi-square test. Abbreviation: MMII, metal mixture inflammatory index; CVD, cardiovascular disease; WWI, weight-adjusted waist index; PIR, poverty income ratio.

### Association between MMII, WWI and CVD

**FIGURE 2:**
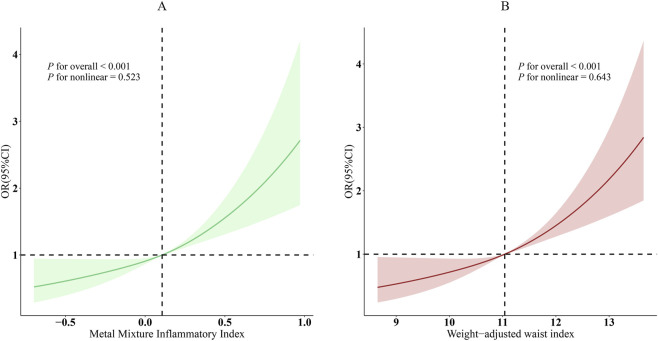
Dose-response relationships between MMII, WWI, and CVD. **(A)**, MMII - CVD; **(B)**, WWI - CVD. OR (solid lines) and 95% confidence levels (shaded areas) were adjusted for age, sex, education level, marital status, PIR, race, diabetes, and hyperlipidemia.

Strong associations were seen between MMII and WWI and prevalent CVD in unadjusted analyses. Each one-unit increase in MMII was associated with higher odds of CVD (OR = 6.49, 95% CI: 4.41–9.55, P < 0.001). Following the adjustment of demographic and clinical covariates, the association weakened, however, it remained statically significant (Model 3: OR = 2.79, 95% CI: 1.81–4.29, P < 0.001). An increase in WWI was linked to higher odds for CVD in both the crude and fully-adjusted model. In crude model, for one-unit increase in WWI, odds of CVD were 2.38 (95% CI: 2.16–2.63, P < 0.001). Similarly, the completely adjusted model showed that WWI was independently associated with CVD, OR = 1.45 (95% CI: 1.25–1.69, P < 0.001). Participants in the highest tertile of WWI had a 66% higher odds of CVD compared with those in the lowest tertile (OR = 1.66, 95% CI: 1.20–2.30; P for trend <0.001) (Table 2). The RCS curve with adjusted model showed the association of MMII, WWI and CVD. According to statistics, MMII and WWI were shown to have significant overall correlation with CVD (P < 0.001), and a statistically significant linear relationship was demonstrated (P for nonlinear >0.05) (Figure 2).

**TABLE 2:** Association between MMII, WWI, and CVD.

Characteristics	Model 1 [OR (95% CI)]	*p-value*	Model 2 [OR (95% CI)]	*p-value*	Model 3 [OR (95% CI)]	*p-value*
MMII - CVD						
Continuous	6.49 (4.41,9.55)	<0.001	2.93 (1.90, 4.51)	<0.001	2.79 (1.81, 4.29)	<0.001
Tertile						
T1	1 (ref.)	​	1 (ref.)	​	1 (ref.)	​
T2	1.71 (1.31,2.22)	<0.001	1.43 (1.05, 1.93)	0.023	1.37 (1.00, 1.86)	0.048
T3	2.90 (2.23,3.77)	<0.001	1.77 (1.32, 2.37)	<0.001	1.70 (1.28, 2.27)	<0.001
*P for trend*	<0.001	​	<0.001	​	<0.001	​
WWI - CVD						
Continuous	2.38 (2.16,2.63)	<0.001	1.69 (1.46, 1.95)	<0.001	1.45 (1.25, 1.69)	<0.001
Tertile						
T1	1 (ref.)	​	1 (ref.)	​	1 (ref.)	​
T2	2.47 (1.80,3.38)	<0.001	1.48 (1.06, 2.07)	0.021	1.22 (0.87, 1.71)	0.244
T3	5.57 (4.29,7.23)	<0.001	2.28 (1.66, 3.15)	<0.001	1.66 (1.20, 2.30)	0.002
*P for trend*	<0.001	​	<0.001	​	<0.001	​

Model 1: No covariates were adjusted. Model 2: age, sex, education level, marital status, PIR, and race were adjusted. Model 3: age, sex, education level, marital status, PIR, race, diabetes, and hyperlipidemia were adjusted. Abbreviation: MMII, metal mixture inflammatory index; CVD, cardiovascular disease; WWI, weight-adjusted waist index; PIR, poverty income ratio; OR, odds ratio; CI, confidence interval.

### Subgroup analysis between MMII, WWI, and CVD

**FIGURE 3:**
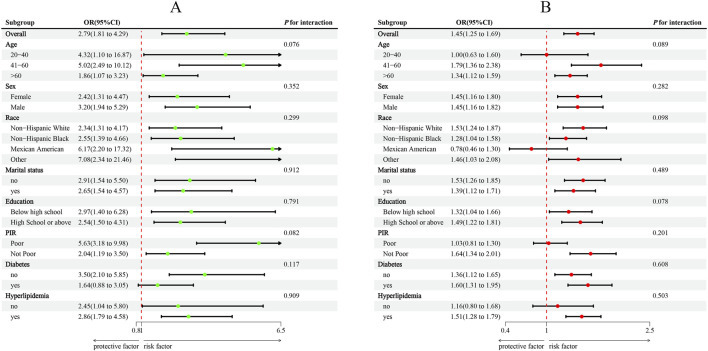
Subgroup analysis between MMII, WWI, and CVD. **(A)**, MMII - CVD; **(B)**, WWI - CVD. ORs were calculated per 1-unit increase in MMII, and each standard deviation increased in WWI. Analyses were adjusted for age, sex, education level, marital status, PIR, race, diabetes, and hyperlipidemia.

Subgroup analyses were conducted for descriptive and exploratory purposes, and formal statistical power to detect interaction effects was limited. Overall, subgroup analyses supported the robustness of the associations among MMII, WWI, and CVD (Figure 3). Younger individuals aged 20–40 years showed a stronger association, with an adjusted odds ratio (OR) of 4.32 (95% confidence interval (CI): 1.10–16.87) whereas the corresponding OR for individuals with middle-aged adults aged 41–60 years was 5.02 (95% CI: 2.49–10.12), compared with older individuals above 60 years, OR is 1.86 (95% CI: 1.07–3.23; P for interaction = 0.076). Associations were also more pronounced among Mexican American participants (OR = 6.17, 95% CI: 2.20–17.32) and those classified as “Other” racial/ethnic groups (OR = 7.08, 95% CI: 2.34–21.46). No significant effect modification was noted by other subgroups. The power to detect interaction effects statistically was limited, as many subgroup analyses were descriptive or exploratory in nature.

### Mediation analyses

**FIGURE 4:**
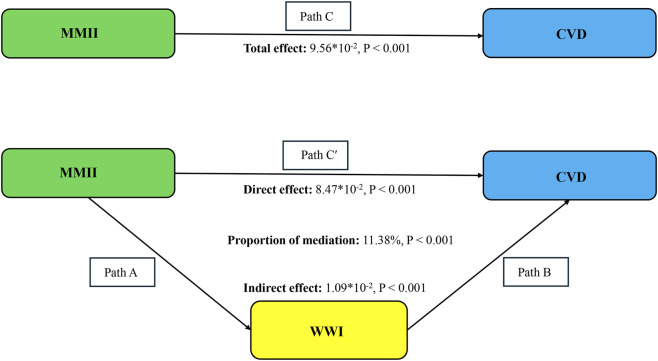
Schematic diagram of the mediation effect analysis. Path C indicates the total effect; path C′ indicates the direct effect. The indirect effect is estimated as the multiplication of paths A and B (path A*B). The mediated proportion is calculated as indirect effect/(indirect effect + direct effect) × 100%. Abbreviation: MMII, Metal Mixture Inflammatory Index; CVD, Cardiovascular Disease; WWI, weight-adjusted waist index. Analyses were adjusted for age, sex, education level, marital status, PIR, race, diabetes, and hyperlipidemia.

Exploratory examinations of mediation revealed WWI statistically accounted for part of the association between MMII and CVD (Figure 4). The overall relationship (Path C: β = 9.56 × 10^−2^, P < 0.001) between MMII and CVD was statistically significant. After inclusion of WWI in the model, the direct association remained statistically significant (Path C′: β = 8.47 × 10^−2^, P < 0.001), while the indirect association via WWI was also significant (β = 1.09 × 10^−2^, P < 0.001). This represented 11.4% of the total association. In light of MMII, WWI, and CVD being assessed at the same time, these mediation findings should be interpreted in an exploratory manner and do not establish a causal pathway (Table 3).

**TABLE 3:** Multivariate linear regression of MMII and WWI.

Exposure – Outcome	β	95%CI	P-value
MMII - WWI	0.20	(0.13, 0.27)	<0.001

Adjusted for age, sex, education level, marital status, PIR, race, diabetes, and hyperlipidemia.

### Association between MMII and mortality

During a median follow-up of 7.3 years (interquartile range: 4.1–10.7 years), a total of 1,134 all-cause deaths and 401 cardiovascular deaths were recorded. Table 4 depicts the relationship between MMII and mortality outcomes. All-cause mortality risk was elevated in participants with a higher MMII level (HR = 2.39, 95% CI: 1.69–3.38, P < 0.001). Individuals in the highest tertile of MMII experienced a 55% increased likelihood of dying from any cause versus those in the lowest tertile (HR = 1.55, 95% CI: 1.27–1.90; P for trend <0.001). When stratified by baseline CVD status, the association between MMII and all-cause mortality was observed among participants without CVD (continuous HR = 2.72, 95% CI: 1.72–4.30; T3 vs. T1 HR = 1.58, 95% CI: 1.22–2.05), but not among those with pre-existing CVD (HR = 1.42, 95% CI: 0.83–2.43). In contrast, MMII was not significantly associated with cardiovascular mortality in the overall population or in subgroup analyses (all P > 0.05).

**TABLE 4:** HRs (95% CIs) are used for all-cause and cardiovascular mortality, according to the MMII.

Characteristics	All-cause mortality [HR (95% CI)]*	*p-value*	Cardiovascular mortality [HR (95% CI)]*	*p-value*
All participants				
Continuous	2.39 (1.69,3.38)	<0.001	0.85 (0.40,1.82)	0.683
Tertile				
T1	1 (ref.)	​	1 (ref.)	​
T2	1.05 (0.83,1.32)	0.692	1.11 (0.71,1.74)	0.650
T3	1.55 (1.27,1.90)	<0.001	1.15 (0.75,1.76)	0.530
*P for trend*	<0.001	​	0.524	​
CVD				
Continuous	1.42 (0.83,2.43)	0.202	0.58 (0.25,1.32)	0.190
Tertile				
T1	1 (ref.)	​	1 (ref.)	​
T2	0.93 (0.61,1.42)	0.740	1.07 (0.60,1.92)	0.820
T3	1.31 (0.91,1.89)	0.150	0.94 (0.57,1.54)	0.810
*P for trend*	0.060	​	0.730	​
Non-CVD				
Continuous	2.72 (1.72,4.30)	<0.001	0.97 (0.27,3.55)	0.970
Tertile				
T1	1 (ref.)	​	1 (ref.)	​
T2	1.07 (0.80,1.44)	0.650	1.09 (0.56,2.13)	0.790
T3	1.58 (1.22,2.05)	<0.001	1.22 (0.62,2.41)	0.560
*P for trend*	<0.001	​	0.540	​

Age, sex, education level, marital status, PIR, race, diabetes, and hyperlipidemia were adjusted. Abbreviation: PIR, poverty income ratio; MMII, metal mixture inflammatory index; CVD, cardiovascular disease; HR, hazard ratio; CI, confidence interval.

## Discussion

In prior epidemiological studies, the individual metals, including cadmium and lead, were associated with higher cardiovascular disease (CVD) and death risks (Lamas et al., 2023; Huang et al., 2024). However, assessing a metal in isolation can underestimate the complexity of real-world exposures, where people are exposed to several different metals that can act additively, synergistically, or antagonistically. To better reflect this complexity, the present study adopted a mixture-oriented approach relied by the current study to better capture this complexity. Specifically, the Metal Mixture Inflammatory Index (MMII) combines multiple urinary metals into one composite with the purpose of representing the overall inflammatory burden of co-exposures. The method provides a better characterization of environmental metal exposure than single-metal studies.

In addition, we examined the weight-adjusted waist index (WWI) as a novel anthropometric indicator in this context. WWI was identified as a strong correlate of both MMII and CVD, and exploratory mediation analyses suggested that WWI statistically accounted for a portion of the association between MMII and CVD. To the best of our knowledge, population-based studies have not previously evaluated whether central obesity mediates the association between metal-related inflammatory burden and cardiovascular outcomes. These findings builds on existing evidence by examining associations between metal mixtures, systemic inflammation, and abdominal adiposity together. Nevertheless, as the majority of data are based on observations and largely cross-sectional, one should regard these results as descriptive and hypothesis generating, not as proof of a causal pathway.

Our findings are consistent with prior toxicological and epidemiological evidence implicating metals such as cadmium, lead, antimony, and uranium in adverse cardiometabolic processes. Experimental and observational studies have shown that exposure to metals is linked to oxidative stress, endothelial dysfunction, and a chronic, low-grade inflammatory response (Jomova et al., 2022; Sun et al., 2022; Gong et al., 2024). By incorporating multiple metals into a single index, MMII captures the overall inflammatory burden of co-exposures and reduces the risk of attributing associations to individual elements in isolation. Along with the above literature, we observed positive relationships of MMII with WWI, and exploratory mediation analyses suggested that central adiposity may represent one of several pathways linking metal-related inflammation to cardiovascular risk. However, these findings should not be taken as evidence of cause and effect. The association observed can be explained by WWI only to some degree which means there could be other possible mechanisms involved. There are said to exist some adiposity-dependent (ectopic fat accumulation and lipotoxic metabolic dysfunction) and adiposity-independent (endothelial injury, autonomic dysregulation and epigenetic modification) mechanisms (Garg et al., 2023; Ahmed et al., 2021).

The observed linear associations between MMII, WWI, and CVD prevalence suggest that increased inflammatory burden related to metal mixtures is associated with higher cardiovascular risk across the distribution of exposure without an apparent threshold. The associations were found to be stronger among the younger and middle-aged adults. Similarly, among the Mexican American and other racial/ethnic groups, the subgroups revealed stronger assessments. These results may indicate variations in exposure sources (diet, occupation, residential area), underlying susceptibility or wider social determinants of health (Erdal et al., 2024; Yang et al., 2021). However, it is important to be cautious about these subgroup results. The analyses were exploratory in nature, the study was not specifically powered to detect interaction effects, and the number of CVD cases within some strata was limited, resulting in wide confidence intervals. In addition, multiple subgroup comparisons increase the likelihood of chance findings. Thus, these findings should not be over-interpreted as evidence of effect modification but merely as hypothesis-generating and warranting verification in larger samples and targeted designs in future research.

In reports of mortality, MMII had a link with all-cause mortality but not cardiovascular. One should be wary of this difference This pattern can possibly be explained in several ways. One possible explanation is that metal mixtures exert systemic toxicity and contribute to a broad range of non-cardiovascular outcomes, including renal, hepatic, respiratory, and neurological diseases, thereby strengthening associations with all-cause mortality while diluting cause-specific cardiovascular associations (Yan et al., 2023; Park and Lee, 2022; Shehata et al., 2023). In addition, the number of cardiovascular deaths in NHANES is relatively limited compared with all-cause mortality, which may reduce statistical power to detect modest associations, particularly in stratified analyses. Misclassification of cause of death based on death certificate data and the presence of competing risks from non-cardiovascular causes may further attenuate associations with cardiovascular mortality (Nakamaru et al., 2023; Zheng et al., 2022). For your information, it simply means that caution is warranted in inferring absence of effect from the null results on cardiovascular mortality. Further studies with longer follow-up and larger number of cardiovascular events are warranted.

The strengths of the current study involve the large-scale utilization of the National Health and Nutrition Examination Survey (NHANES) study, standardization of data collection methods, control of laboratory quality, and creatinine adjustment of urinary metal measurements. The application of a data-driven mixture index (MMII) enabled assessment of cumulative inflammatory burden related to multi-metal exposure, while the inclusion of WWI provided additional insight into the role of central adiposity. Multiple analytic approaches were applied, including survey-weighted regression models, restricted cubic splines, subgroup analyses, and exploratory mediation analyses. Although the MMII demonstrated consistent associations across models, external validation in independent populations is needed to confirm its generalizability.

Several limitations should also be acknowledged. First, MMII, WWI, and CVD status were assessed within a largely cross-sectional framework; therefore, temporality cannot be established, and reverse causation cannot be excluded. Accordingly, mediation analyses should be interpreted as exploratory and hypothesis-generating rather than causal. Second, exposure misclassification may have been generated in some cases since urinary metals were measured only at a single time point. Third, despite adjustment for multiple sociodemographic and clinical covariates, residual confounding may remain. Fourth, CVD status was based on self-reported physician diagnoses rather than adjudicated clinical records, which may have introduced non-differential misclassification. Fifth, the number of cardiovascular deaths was relatively limited, potentially reducing statistical power for cause-specific mortality analyses. Finally, although survey weights were applied in the main analyses, mediation models were not survey-weighted due to current methodological limitations, which may affect generalizability; similar analytic choices have been used in prior NHANES-based mediation studies. Moreover, analysis was limited to participants with complete MMII-related data. If excluded participants possess relevant differences, this may introduce selection bias.

## Conclusion

In this nationally representative NHANES study (n = 11,577), wwe used Metal Mixture Inflammatory Index (MMII) to examine the burden of cumulative inflammation due to exposure to a number of metals. Higher MMII levels were associated with increased all-cause mortality, whereas no statistically significant association was observed with cardiovascular mortality. In addition, the weight-adjusted waist index (WWI) was positively associated with CVD prevalence, and exploratory mediation analyses suggested that central obesity statistically accounted for a portion of the association between MMII and CVD. Collectively, these findings support the relevance of mixture-based approaches in environmental epidemiology and suggest that abdominal obesity may represent an important correlate in the relationship between metal-related inflammatory burden and adverse health outcomes. Although causal inferences cannot be drawn from this observational analysis, our results highlight the potential value of integrated research strategies that jointly consider environmental exposures, systemic inflammation, and metabolic health in understanding cardiometabolic risk at the population level.

## Data Availability

The original contributions presented in the study are included in the article/Supplementary Material, further inquiries can be directed to the corresponding authors.
