# Correction: Cumulative inflammatory burden of metal mixtures is associated with central obesity, cardiovascular disease, and mortality: findings from NHANES

**DOI:** 10.3389/fcell.2026.1817293

**Published:** 2026-03-05

**Authors:** Frontiers Production Office

**Affiliations:** Frontiers Media SA, Lausanne, Switzerland

**Keywords:** metal mixtures, inflammatory burden, cardiovascular disease, central obesity, NHANES, mortality risk

Affiliation 1 Department of General Surgery, The Sihui People’s Hospital, Zhaoqing, China was erroneously given as Department of General Surgery, Zhaoqing Central People’s Hospital, Zhaoqing, China.

Author Yiduo Bai was erroneously assigned to affiliation 2. This affiliation has now been updated to 3 for author Yiduo Bai.

Author Wenjun Liu was erroneously assigned to affiliation 2. This affiliation has now been updated to 3 for author Wenjun Liu.

Author Jijun Wu was erroneously assigned to affiliation 4. This affiliation has now been updated to 5 for author Jijun Wu.

The original version of this article has been updated.

