# Supplementary material for: Cumulative inflammatory burden of metal mixtures is associated with central obesity, cardiovascular disease, and mortality: findings from NHANES
Source: Front Cell Dev Biol. 2026 Feb 18;14:1717247. doi: 10.3389/fcell.2026.1717247 (PMC12956676; doi:10.3389/fcell.2026.1717247)
Supplement: Supplementary file 1 [file Table1.docx]

**Supplementary Material**

**Table S1**

**The detection limits and detection rates of urine heavy metals.**

| Metals (μg/L) | LOD (range) | Detection rate (%) |
| --- | --- | --- |
| Mercury | 0.08-0.12 | 94.15 |
| Cadmium | 0.04-0.05 | 94.26 |
| Cobalt | 0.04-0.05 | 99.45 |
| Molybdenum | 0.91-2.28 | 99.93 |
| Lead | 0.09-0.10 | 97.32 |
| Platinum | 0.00-0.01 | 9.68 |
| Antimony | 0.03-0.04 | 73.72 |
| Thallium | 0.01-0.02 | 99.49 |
| Tungsten | 0.02-0.03 | 88.35 |
| Uranium | 0.00-0.01 | 89.89 |

Abbreviation: LOD: limit of detection

**Table S2**

Definition of variables involved in this study.

| Variables | Description in NHANES |
| --- | --- |
| Age | Divided into three groups: 20-40 years old, 41-60  years old, >60 years old |
| Sex | Male and Female |
| Race | Mexican American, Non-Hispanic Black, Non-Hispanic White, Other Race |
| Educational level | Below high school, High School or above |
| Marital status | Yes: Married/Living with partner |
| PIR | Poor: <1.3; Not Poor:>=1.3 |
| Diabetes | Diabetes was defined as a history of previous diabetes, HbA1c level ≥6.5%, or fasting blood glucose level ≥126 mg/dL |
| Hyperlipidemia | (1) Triglyceride (TG) levels ≥150 mg/dl (1.7 mmol/L);(2) Total cholesterol (TC) levels ≥200 mg/dl (5.18 mmol/L);(3) Low-density lipoprotein (LDL) levels ≥130 mg/dl (3.37 mmol/L);(4) High-density lipoprotein (HDL) levels: Men: <40 mg/dl (1.04 mmol/L); Women: <50 mg/dl (1.30 mmol/L) ;(5) Individuals taking cholesterol-lowering drugs are also considered hyperlipidemia. |

PIR, poverty income ratio

**Table S3 Details of reduced rank regression.**

Reduced rank regression is a multivariate statistical method that achieves dimensionality reduction while using multiple independent variables to predict or explain multiple dependent variables. RRR works essentially by finding linear combinations of the predictors (factors) to use to predict the responses linearly. Based on the assumption of traditional multivariate linear regression, it restricts the rank of the regression coefficient matrix.

The goal of ordinary least squares (OLS) is to minimize the sum of squared residuals, that is, to solve the regression coefficient matrix to minimize the following equation:

L = ||Y - XB||^2^

Assuming the number of independent variables is p, the number of dependent variables is q, the sample size is n, X is the independent variable matrix (n × p), Y is the dependent variable matrix (n × q), B is the regression coefficient matrix (p × q), L is the loss function.

RRR restricts the rank of the regression coefficient matrix, requiring that the sum of squared residuals be minimized under the premise of m ≤ q (m is the rank of B). The first factor of RRR explains more variation in response than any other linear function of predictors ^[1]^

[1]. Hoffmann, K., et al., Application of a new statistical method to derive dietary patterns in nutritional epidemiology. Am J Epidemiol, 2004. 159(10): p. 935-44.
